# INFLAMMATORY DIET PATTERN AND COGNITIVE FUNCTION IN 5 EUROPEAN COUNTRIES OVER 3-YEARS FOLLOW-UP

**DOI:** 10.1093/geroni/igz038.3343

**Published:** 2019-11-08

**Authors:** Patricia O Chocano-Bedoya, Bruno Vellas, Reto W Kressig, Endel J Orav, Walter Willett, Heike A Bischoff-Ferrari

**Affiliations:** 1 Centre on Aging and Mobility, University of Zurich and City Hospital Waid, Zurich, Switzerland; 2 Institut des Sciences du Cerveau, de la Cognition et du Comportement de Toulouse (ISCT), Toulouse, France; 3 Felix Platter Hospital · University Center for Medicine of Aging Basel, Switzerland, Basel, Switzerland; 4 Department of Biostatistics, Harvard T.H. Chan School of Public Health, Boston, Massachusetts, United States; 5 Department of Nutrition, Harvard T.H. Chan School of Public Health, Boston, Massachusetts, United States

## Abstract

Diet patterns associated with low chronic inflammation may modulate cognitive decline. We investigated an empirical dietary pattern (EDP) associated with inflammation in five European countries and its association with cognitive changes over 3-years. This prospective study included 2157 community dwelling-seniors 70 years and older, followed for 3 years as part of DO-HEALTH, a randomized clinical trial. At baseline, participants completed a food frequency questionnaire and C-Reactive Protein (CRP) and interleukin-6 (IL-6) was measured. We used the Montreal Cognitive Assessment (MoCA) every year of the study. Based on reduced rank regression, we estimated a dietary pattern associated with CRP and IL-6. To evaluate the association between the EDP and cognitive changes over time, we used repeated measure linear regression models adjusting for age, total calories, BMI, study center, time, alcohol intake, education, physical activity, presence of depression symptoms, hypertension, diabetes or heart disease. The EDP was characterized by higher intakes of red and organ meat, refined grains, legumes, poultry and white fish, and lower intakes of coffee, tea, ginger, nuts and cheese. In multivariate adjusted models, participants with lowest adherence to the EDP (range -7.3 to -0.3) increased their MoCA scores 0.7 points over three years whereas those with highest adherence (range 0.4-10.1) increased their MoCA scores only by 0.2 points (p=0.01). In conclusion, a low inflammatory diet was associated with better cognitive function over time among adults ≥70 years from five European countries. This finding supports the role of diet in the promotion of cognitive health among older adults.

